# Inherited Disorders of Iron Overload

**DOI:** 10.3389/fnut.2018.00103

**Published:** 2018-10-29

**Authors:** Kostas Pantopoulos

**Affiliations:** ^1^Lady Davis Institute for Medical Research, Jewish General Hospital, Montreal, QC, Canada; ^2^Department of Medicine, McGill University, Montreal, QC, Canada

**Keywords:** hemochromatosis, hepcidin, aceruloplasminemia, ferroportin, hypotransferrinemia, HFE, hemojuvelin (HJV), transferrin receptor 2 (TFR2)

## Abstract

Dietary iron absorption and systemic iron traffic are tightly controlled by hepcidin, a liver-derived peptide hormone. Hepcidin inhibits iron entry into plasma by binding to and inactivating the iron exporter ferroportin in target cells, such as duodenal enterocytes and tissue macrophages. Hepcidin is induced in response to increased body iron stores to inhibit further iron absorption and prevent iron overload. The mechanism involves the BMP/SMAD signaling pathway, which triggers transcriptional hepcidin induction. Inactivating mutations in components of this pathway cause hepcidin deficiency, which allows inappropriately increased iron absorption and efflux into the bloodstream. This leads to hereditary hemochromatosis (HH), a genetically heterogenous autosomal recessive disorder of iron metabolism characterized by gradual buildup of unshielded non-transferrin bound iron (NTBI) in plasma and excessive iron deposition in tissue parenchymal cells. The predominant HH form is linked to mutations in the *HFE* gene and constitutes the most frequent genetic disorder in Caucasians. Other, more severe and rare variants are caused by inactivating mutations in *HJV* (hemojuvelin), *HAMP* (hepcidin) or *TFR2* (transferrin receptor 2). Mutations in *SLC40A1* (ferroportin) that cause hepcidin resistance recapitulate the biochemical phenotype of HH. However, ferroportin-related hemochromatosis is transmitted in an autosomal dominant manner. Loss-of-function ferroportin mutations lead to ferroportin disease, characterized by iron overload in macrophages and low transferrin saturation. Aceruloplasminemia and atransferrinemia are further inherited disorders of iron overload caused by deficiency in ceruloplasmin or transferrin, the plasma ferroxidase and iron carrier, respectively.

## Iron homeostasis

**Figure 1:**
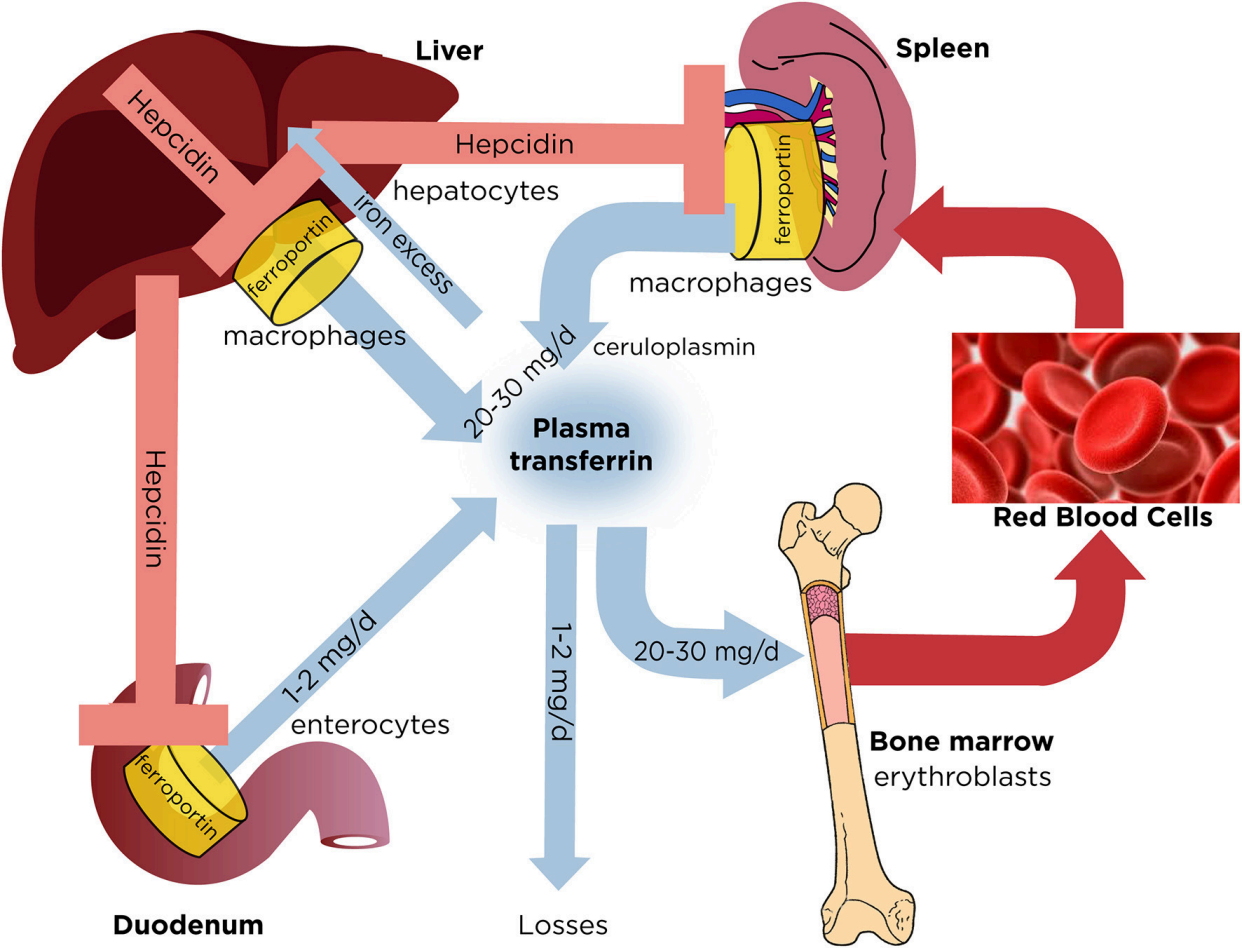
Dynamics of systemic iron balance. Plasma transferrin delivers iron to bone marrow erythroblasts and to other tissues. It contains a very small (~0.1%) but highly dynamic fraction of body iron that turns over >10 times/day to meet the iron need for erythropoiesis (20–30 mg/day). The transferrin iron pool is primarily replenished with iron recycled from hepatic and splenic macrophages during erythrophagocytosis of senescent red blood cells. Duodenal enterocytes absorb dietary iron and release small amounts (1–2 mg/day) to compensate for non-specific losses. Hepatocytes store excess of body iron, which can be mobilized to plasma under iron deficiency. Iron efflux to plasma from macrophages, enterocytes or hepatocytes is negatively regulated by hepcidin, a liver-derived peptide hormone that binds to the iron exporter ferroportin and promotes its degradation.

Iron is an essential component for almost all living cells and organisms. However, when present in excess, iron becomes a potential biohazard due to its redox reactivity that promotes oxidative stress. Thus, balanced iron metabolism is imperative for health and its deregulation leads to disease (1). In mammals, the vast majority of body iron (>70%) is distributed in red blood cells and mediates oxygen transport within hemoglobin (Figure 1). Tissue macrophages clear senescent red blood cells and recycle iron to erythroblasts for re-utilization. Iron release into plasma involves ferroportin, the sole ferrous (Fe^2+^) iron exporter. Following its export, ferrous iron undergoes oxidation to ferric (Fe^3+^) by ceruloplasmin, a circulating ferroxidase, and captured by the iron carrier transferrin. The main function of transferrin is iron delivery to tissues via *transferrin receptor 1* (TfR1). Transferrin is also critical for keeping plasma iron in a redox inert state; thus, under physiological conditions it is only saturated by ~30% with iron, and iron-free apo-transferrin possess a high iron buffering capacity (2). Iron-loaded holo-transferrin contains a small (~0.1%) but highly dynamic fraction of body iron that turns over >10 times/day to satisfy the high erythropoietic requirements. In humans, these range between 20 and 30 mg of iron per day (3). Dietary iron absorption (1–2 mg/day in adults) mainly serves to compensate non-specific iron losses and minimally contributes to the transferrin iron pool under physiological conditions. Duodenal enterocytes internalize iron from the intestinal lumen via the *divalent metal transporter 1* (DMT1), end export it to plasma via ferroportin.

Iron entry into the bloodstream is critical for systemic iron homeostasis and is negatively regulated by hepcidin, the iron regulatory hormone (4). Hepcidin is expressed in hepatocytes as a pre-pro-peptide and undergoes proteolytic processing. The mature bioactive hormone is a cysteine-rich peptide of 25 amino acids. Hepcidin operates by binding to ferroportin in tissue macrophages, duodenal enterocytes and other target cells (Figure 1). This triggers ubiquitination, internalization and degradation of ferroportin in lysosomes (5). As a result, iron is sequestered within macrophages, dietary iron absorption is inhibited, and plasma iron levels drop. These are physiological responses to iron intake or inflammation and are mediated by hepcidin. In fact, iron and inflammation are major hepcidin inducers. Following iron intake or an increase in body iron stores, hepcidin is mainly upregulated to prevent further dietary iron absorption. Under inflammatory conditions hepcidin induction serves to promote hypoferremia and iron sequestration in macrophages, presumably as an innate immune response to deprive invading bacteria from essential iron (6). Iron deprivation strategies of the host are known as *nutritional immunity* (7).

## Regulation of hepcidin

**Figure 2:**
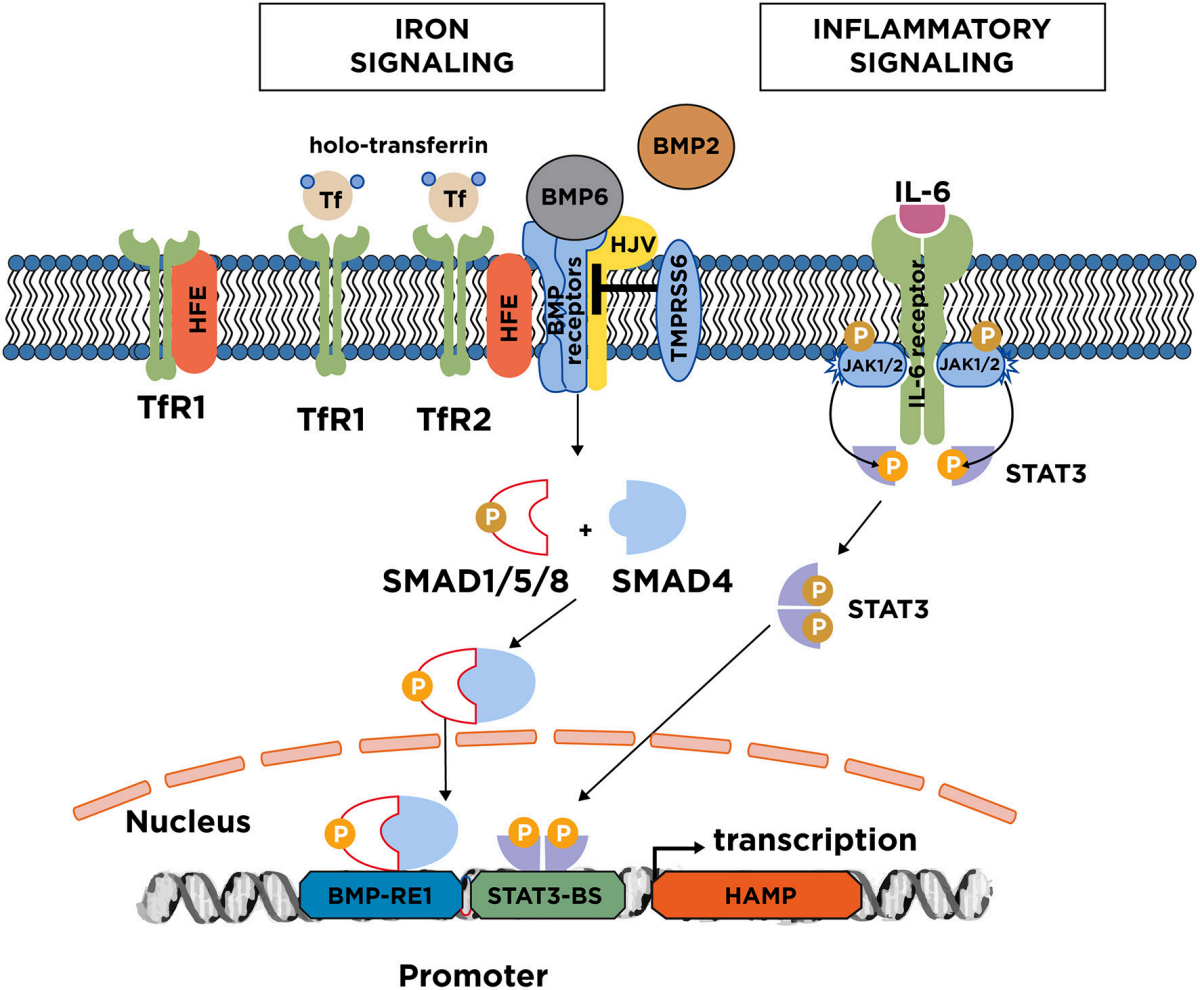
Iron and inflammatory signaling to hepcidin. Increases in serum or tissue iron promote transcriptional induction of hepcidin via the BMP/SMAD signaling pathway. Key upstream events are an increase in transferin saturation and the secretion of BMP6, and to a lesser extent BMP2 from liver sinusoidal endothelial cells. BMP6 binds to type I and II receptors on the surface of hepatocytes. With the critical aid of auxiliary factors, such as HJV, HFE, and TfR2, this leads to phosphorylation of regulatory SMAD1/5/8, recruitment of SMAD4, and translocation of the complex to the nucleus for binding to the hepcidin promoter. TfR2 likely operates as plasma iron sensor; TfR1 is thought to mediate uptake of transferrin-bound iron but can also negatively affect iron signaling by sequestering HFE. Iron signaling to hepcidin is negatively regulated by the serine protease matriptase-2 (TMPRSS6), which cleaves and inactivates components of the signaling complex. The major inflammatory signaling pathway to hepcidin involves IL-6, which binds to IL-6 receptors on hepatocytes. This promotes dimerization of the receptors and activation of associated JAK1/2, which in turn phosphorylate STAT3. Subsequently, phospho-STAT3 dimerizes and translocates to the nucleus for binding to the hepcidin promoter.

Acute and chronic iron loading are thought to promote hepcidin induction by distinct mechanisms (8, 9). The key upstream events appear to be an increase in iron saturation of plasma transferrin, and the iron-dependent secretion of *bone morphogenetic protein 6* (BMP6) from liver sinusoidal endothelial cells (10). These cells also secrete BMP2, which is less responsive to alterations in iron levels and may control basal hepcidin expression (11, 12). The binding of BMP6 or BMP2 to type I (ALK2 and ALK3) and type II (ActRIIA and BMPR2) BMP receptors on the plasma membrane of hepatocytes promotes phosphorylation of regulatory SMAD1/5/8, recruitment of SMAD4, and translocation of the complex to the nucleus for transcriptional activation of the hepcidin (HAMP) promoter (Figure 2). The BMP/SMAD signaling pathway requires upstream auxiliary factors: (a) Hemojuvelin (HJV), a BMP co-receptor; (b) The hemochromatosis protein HFE, an atypical *major histocompatibility complex class 1* (MHC class 1) type molecule; and (c) *Transferrin receptor 2* (TfR2), a sensor of iron-loaded plasma transferrin. The cascade is negatively regulated by the serine protease matriptase-2 (TMPRSS6), which cleaves and thereby inactivates the type I and II BMP receptors, HFE, HJV, TfR2 and possibly additional cofactors (13). HFE can specifically interact with TfR1 (14), which appears to limit its signaling function (15).

Inflammation triggers hepcidin induction via the IL-6/STAT3 pathway (Figure 2). Following its secretion from activated macrophages, the inflammatory cytokine IL-6 binds to the IL-6 receptor on hepatocytes. This promotes phosphorylation of STAT3 by JAK1/2 kinases, and translocation of phospho-STAT3 to the nucleus for transcriptional activation of the HAMP promoter. Further cytokines, such as IL-1β (16), IL-22 (17), or IFNα (18) contribute to inflammatory hepcidin induction by additional pathways. Experimental evidence suggests that the hepcidin-dependent hypoferremic response to acute inflammation requires a threshold of BMP6/HJV/SMAD signaling (19).

Hepcidin is also regulated by additional positive or negative stimuli (20). Hepcidin suppression occurs under iron deficiency or hypoxemia, and serves to facilitate iron mobilization for erythropoiesis (3). This is mediated by erythroferrone (ERFE), a hormone secreted by erythroblasts in response to erythropoietin (21) that antagonizes BMP6-mediated hepcidin induction (22).

## Hereditary hemochromatosis

Hemochromatosis was first described in 1865 by the French physician Armand Trousseau as “bronze diabetes” (23). The name refers to the pronounced skin pigmentation in some patients presenting with diabetes. Nevertheless, Trousseau did not associate this phenotype with iron overload. This was done in 1890 by the German pathologist Friedrich Daniel von Recklinghausen, who first introduced the term “hemochromatosis” (from Greek αíμα = blood and $\chi \rho \overset{´}{\omega }\mu \alpha$ = color) to associate the disease with skin pigmentation, and suggested that iron overload may disrupt endocrine functions of the pancreas (24). The hereditary nature of hemochromatosis was demonstrated by Marcel Simon and colleagues in the 1970's (25, 26).

Today, we understand hereditary hemochromatosis as a genetically heterogenous endocrine disease of iron overload that varies in molecular etiology and clinical presentation. The common denominator in the disease subtypes is a mild or severe disruption of the hepcidin pathway due to mutations in genes encoding auxiliary factors in BMP/SMAD iron signaling to hepcidin. This results in insufficient hepcidin responses to iron intake or to high body iron stores, and causes loss of hepcidin-mediated feedback inhibition in dietary iron absorption (27). The extent of hepcidin inhibition correlates with the degree of systemic iron overload.

The pathogenesis of hereditary hemochromatosis involves uncontrolled iron absorption and efflux into the bloodstream (at a rate up to 8–10 mg/day), gradually leading to oversaturation of transferrin and buildup of non-transferrin-bound iron (NTBI). Unshielded NTBI, which is redox-active and toxic, is eventually taken up by tissue parenchymal cells, especially in the liver, pancreas and heart (28). Hepatocytes and pancreatic acinar cells internalize NTBI via the transporter SLC39A14 (or ZIP14) and become iron overloaded (29). Paradoxically, tissue macrophages and duodenal enterocytes become iron-deficient in spite of systemic iron overload. These cells fail to retain iron because they overexpress ferroportin as a result of hepcidin insufficiency.

If untreated, hereditary hemochromatosis has the potential to cause multiorgan damage and failure. In adult hemochromatosis, clinical complications usually manifest after the fourth decade of life. The most serious of them is liver fibrosis, which may progress to cirrhosis and hepatocellular cancer (30, 31). Other complications include type 1 diabetes mellitus (32), arthropathy (33) and osteoporosis (34). In early-onset juvenile hemochromatosis, the most severe clinical complications are cardiomyopathy (35) and hypogonadism (36).

The standard treatment of all adult and juvenile hemochromatosis patients is therapeutic phlebotomy (37). This directly reduces iron burden since one unit (400–500 ml) of blood contains ~200–250 mg of iron and, moreover, promotes iron mobilization from stores. There is no evidence from clinical trials for optimal protocol of therapeutic phlebotomy regarding start time of the treatment, frequency, duration and endpoint. Current recommendations are empirical (38, 39). Weekly phlebotomy can restore normal plasma iron levels within 1–2 years. Maintenance therapy, which typically involves removal of 2–4 units/year for adult and 6–8 units/year for juvenile hemochromatosis, must be continued lifelong. Survival of phlebotomized patients without liver cirrhosis and type 1 diabetes is equivalent to that of the normal population (40). However, phlebotomy cannot reverse liver cirrhosis, type 1 diabetes, destructive arthritis, cardiomyopathy or hypogonadism. Another drawback is that some patients are intolerant or have low compliance to life-long treatment. Iron chelation therapy may be useful but is not widely used for the treatment of hemochromatosis. Hepcidin replacement therapy could provide an etiologic cure; thus, the interest on the development of hepcidin therapeutics is growing (41).

## Molecular types of hereditary hemochromatosis

### HFE hemochromatosis

HFE hemochromatosis (often classified as type 1; OMIM #235200) is associated with mutations in the HFE gene and constitutes the most frequent form of hereditary hemochromatosis, with high prevalence in populations of Northern European ancestry. It is transmitted in an autosomal recessive fashion. The search for a hemochromatosis gene was initiated as soon as the genetic nature of the disease became clear (25, 26). The *HFE* (high Fe) gene was first discovered as such in 1996 by linkage disequilibrium and haplotype analysis from a large patient cohort (42). It is located on the short arm of chromosome 6 (6p21.3) and encodes an atypical MHC class I protein consisting of three extracellular subunits (α1, α2, and α3), a transmembrane domain and a cytoplasmic tail. Similar to other MHC class 1 proteins, HFE interacts with β_2_-microglobulin, which enables its intracellular processing and translocation to the cell surface. HFE cannot efficiently present antigen peptides to immune cells because the groove between its α1 and α2 subunits is smaller compared to that of typical MHC class I proteins (43). Nevertheless, it possesses limited immunological functions (44).

A C282Y substitution, which abrogates the binding of HFE to β_2_-microglobulin is the most common disease-associated mutation. It first appeared in Northern Europe before 4,000 BC during the transition from iron-rich meat-based diets obtained by hunting to iron-poor cereal-based diets provided from agriculture. The C282Y HFE mutation spread quickly, presumably because it conferred advantage in iron acquisition (45), and possibly also resistance to infection with intracellular pathogens that grow within macrophages, such as *S. typhi* or *M. tuberculosis* (46). Homozygosity for the C282Y HFE mutation is 1:200, but the clinical penetrance is < 30% in males and ~1% in females (47). This suggests that C282Y is not pathogenic *per se*, but rather a polymorphism that predisposes to iron overload in conjunction with gender, alcohol consumption, and genetic/epigenetic factors. Nevertheless, targeted disruption of Hfe (48) or replacement of wild type Hfe (49) with the C282Y ortholog in mice results in an iron overload phenotype, with variable degree among different genetic backgrounds (50, 51). Genetic studies in patients and mouse models identified several genes that appear to modulate the penetrance of HFE hemochromatosis. These include *HAMP* (52), *BMP2, BMP4, HJV* (53), *BMP6* (54), *TMPRSS6* (55), *CYBRD1* (56), *CP* (57), *TF* (58), *GNPAT* (59), *HP* (60).

Several lines of evidence suggest that HFE operates as an upstream regulator for iron signaling to hepcidin in hepatocytes via the BMP/SMAD pathway. Thus, HFE hemochromatosis patients (61) and Hfe^−/−^ mice (62) express inappropriately low hepcidin levels and exhibit blunted hepcidin responses to iron intake (8, 63) due to defective BMP/SMAD signaling (64, 65). Hepatocyte-specific disruption of Hfe recapitulates hepcidin suppression and iron overload in mice (66). Co-ablation of Hfe does not aggravate iron overload in Hjv^−/−^ mice, suggesting that Hfe acts in the same pathway with the BMP co-receptor Hjv (67). Consistent biochemical studies suggested that HFE operates by stabilizing the type I BMP receptor ALK3 (68).

### Juvenile hemochromatosis

Juvenile hemochromatosis (type 2) is an early onset recessive form of hereditary hemochromatosis. Iron overload and related clinical complications develop in the late teens to early twenties. The disease is rare and most affected families were documented in Greece, Southern Italy, and the Saguenay region of Quebec, Canada. Juvenile hemochromatosis was first mapped to chromosome 1q21 (69) but further studies uncovered genetic heterogeneity. Thus, another form of the disease, with indistinguishable clinical phenotype, was found unrelated to the 1q chromosome (70). Subsequent work demonstrated that the relatively more predominant 1q-linked form of juvenile hemochromatosis (subtype 2A; OMIM #602390) is caused by inactivation of the *HJV* gene encoding hemojuvelin that severely suppresses hepcidin expression (71), while the 1q-unlinked form (subtype 2B; OMIM #613313) is directly caused by inactivation of the *HAMP* gene encoding hepcidin (72). Hjv^−/−^ (73, 74) and Hamp^−/−^ (75, 76) mice, as well as hepatocyte-specific Hjv^−/−^ (77, 78) and Hamp^−/−^ (79) mice recapitulate severe iron overload and represent mouse models of juvenile hemochromatosis.

The identification of *HJV* and *HAMP* as juvenile hemochromatosis genes led to the discovery of HJV as a BMP co-receptor, and the establishment of the major role of the BMP/SMAD signaling pathway in hepcidin regulation (80). Later on, BMP6 was shown to be a key upstream inducer of hepcidin in hepatocytes (81, 82). HJV operates as an enhancer of BMP/SMAD signaling and the most frequent pathogenic HJV mutation, a G320V substitution, largely abrogates this function (80). Hjv^−/−^ mice retain iron-dependent regulation of hepcidin, albeit at minuscule residual levels (83).

### TfR2 hemochromatosis

TfR2 hemochromatosis (type 3; OMIM #604250) is linked to inactivation of the *TFR2* gene on chromosome 7q22, which encodes *transferrin receptor 2* (TfR2). It was first described in patients bearing a homozygous non-sense mutation in TfR2 (Y250X) (84) and was later found to be associated with hepcidin suppression (85). The disease is recessive, and the clinical phenotype varies as to the age of onset and severity. It is usually intermediate compared to the phenotypes of HFE-related and juvenile hemochromatosis, but some patients may present with early iron overload (86). Tfr2^−/−^ (87, 88) and hepatocyte-specific Tfr2^−/−^ (89) mice offer models of TfR2 hemochromatosis.

Compound inactivation of TfR2 and HFE in a patient has been associated with juvenile hemochromatosis (90). Likewise, double Tfr2^−/−^Hfe^−/−^ mice manifest more severe iron overload compared to single Tfr2^−/−^ or Hfe^−/−^ littermates (91), suggesting that TfR2 and HFE exhibit non-overlapping functions. Nevertheless, both TfR2 and HFE are required for appropriate BMP/SMAD signaling in mice (65). Conceivably, TfR2 operates as a sensor of transferrin-bound iron, which as a known signal for hepcidin (8, 9). TfR2 is specifically expressed in hepatocytes and erythroid cells. Unlike TfR1, which defines the major entry point of iron into cells, TfR2 appears to primarily function as a sensor of circulating iron that coordinates systemic iron traffic via hepcidin with iron utilization in erythropoiesis (92).

## Ferroportin hemochromatosis

*Ferroportin hemochromatosis* (OMIM #606069) is caused by gain-of-function mutations in the ferroportin-encoding *SLC40A1* gene on chromosome 2q32, leading to hepcidin resistance (93). Although it has similar clinical features and phenotypic hallmarks to other forms of hereditary hemochromatosis (high transferrin saturation, parenchymal iron overload, macrophage iron deficiency), it is transmitted in an autosomal dominant fashion. Therefore, ferroportin hemochromatosis represents a distinct disease entity. The first reported case was linked to the N144H substitution (94). A strong ferroportin gain-of-function phenotype is caused by point mutations at C326, a residue that directly interacts with hepcidin. Patients bearing a ferroportin C326S substitution develop early onset iron overload (95) and express high levels of hepcidin (96). Engineered ferroportin C326S mice develop severe iron overload and represent an animal model for ferroportin hemochromatosis (97). However, contrary to human patients, ferroportin C326S mice develop exocrine pancreatic failure, which reduces their life span to 7–14 months. This phenotype is linked to excessive iron accumulation in pancreatic acinar cells, but manifests exclusively in ferroportin C326S mice and not in humans or other mouse models of hemochromatosis. The reason for this is unclear.

## Ferroportin disease

The ferroportin disease (OMIM #606069) is an autosomal dominant disorder caused by loss-of-function mutations in the ferroportin-encoding *SLC40A1* gene (93). It is more frequent than juvenile or TfR2 hemochromatosis. Patients develop moderate to severe iron overload, mostly in tissue macrophages but also parenchymal cells, which is associated with low transferrin saturation and serum iron. They exhibit reduced tolerance to therapeutic phlebotomy, which can lead to anemia, in spite of persistently elevated serum ferritin levels. An Italian pedigree with the above clinical, biochemical and genetic manifestations was first described in 1999 (98). The disease was subsequently causatively associated with a ferroportin A77D substitution, which impairs its iron export function (99). Patients with loss-of-function ferroportin mutations overexpress hepcidin (100). The flatiron mouse, carrying a ferroportin H32R substitution, represents a model of ferroportin disease (101).

The molecular basis of ferroportin disease has always been puzzling. Recent biochemical data provide a potential explanation to the seemingly paradoxical development of iron overload in patients with defective dietary iron absorption (102). According to this model, wild type ferroportin generated by the unaffected allele traffics as a monomer to the basolateral membrane of intestinal enterocytes and pumps low levels of dietary iron into the circulation. However, wild type ferroportin fails to reach the membrane in macrophages, at least at levels to satisfy the extremely high iron turnover by these cells, which results in macrophage iron retention.

The disorders caused by loss-of-function A77D (99) or gain-of-function N144H (94) ferroportin mutations were described at approximately the same time. Since then, further disease-associated ferroportin missense mutations and deletions have been reported (93). There has been some confusion on the nomenclature and classification of the ferroportin-related disorders, which can be found in the literature as “type 4 hemochromatosis” or “ferroportin-disease” and are registered in the *Online Mendelian Inheritance in Man* (OMIM) database under a common reference number (OMIM #606069). A distinction was initially made between the phenotypes caused by loss- or gain-of-function ferroportin mutations, which were designated as “subtype 4A or 4B hemochromatosis,” or “type A or B ferroportin disease,” respectively. Nevertheless, this classification is not accurate due to the distinct clinical and biochemical characteristics of these entities. Herein, we follow the nomenclature proposed by A. Pietrangelo (93), naming the disorder caused by gain-of-function ferroportin mutations “ferroportin hemochromatosis,” and the disorder caused by loss-of-function ferroportin mutations “ferroportin disease.”

## Aceruloplasminemia

Congenital aceruloplasminemia (OMIM #604290) is a rare recessive disorder caused by loss of ceruloplasmin function due to mutations in the *CP* gene on chromosome 3q23-q24 (103). Ceruloplasmin is secreted from hepatocytes into the bloodstream and its ferroxidase activity serves to facilitate iron efflux from macrophages via ferroportin. An alternatively spliced glycosylphosphatidylinositol (GPI)-anchored form of ceruloplasmin is expressed in astrocytes and plays an important role in brain iron traffic. Even though forms of hypoceruloplasminemia, with partial ceruloplasmin expression, were known earlier (104), the first case of aceruloplasminemia was reported in a 52-years old Japanese female presenting with blepharospasm, retinal degeneration and diabetes mellitus (105). This clinical phenotype was later attributed to a frameshift mutation in the *CP* gene leading to complete loss of ceruloplasmin expression (106). The association between aceruloplasminemia and systemic iron overload was demonstrated at the same time (107).

Aceruloplasminemia patients exhibit low transferrin saturation and tend to develop mild microcytic anemia. Nevertheless, they accumulate excessive iron deposits in parenchymal and non-parenchymal cells of visceral organs (liver, pancreas, spleen) but also in the brain, which is the hallmark of the disease. Aceruloplasminemia is the only inherited disorder of iron metabolism with simultaneous systemic and brain iron overload and is considered a neurodegenerative disorder.

The combination of low serum iron and peripheral tissue iron overload in aceruloplasminemia is similar to that observed in ferroportin disease and can be explained by the defective iron mobilization from tissue macrophages due to lack of ceruloplasmin. Iron overload in the CNS is very likely linked to the loss of GPI-ceruloplasmin from astrocytes, which deregulates brain iron traffic. Iron chelation with oral chelators (108), ceruloplasmin replacement therapy (109) or combination of these approaches (110) appear to improve neurological symptoms. Cp^−/−^ mice bearing targeted disruption of ceruloplasmin represent a model of aceruloplasminemia (111, 112).

## Atransferrinemia

Congenital atransferrinemia (OMIM #209300) is a rare, early onset recessive disorder caused by transferrin deficiency (< 20 mg/dl) due to mutations in the transferrin-encoding *TF* gene on chromosome 3q22.1. The disease is also referred to as hypotransferrinemia, as the complete absence of functional transferrin is lethal. Patients exhibit very low to undetectable levels of plasma transferrin (113). This leads to impaired erythropoiesis, microcytic hypochromic anemia, growth retardation and iron overload in parenchymal cells of the liver, heart and pancreas. The first reported patient was a 7-years old girl with traces of plasma transferrin and systemic iron overload that led to early death due to congestive heart failure (114). Most characterized patients are compound heterozygotes harboring missense or nonsense mutations in the *TF* gene. They exhibit high saturation of residual transferrin with iron and accumulate NTBI. Hepcidin is suppressed due to increased erythropoietic drive (115), which is consistent with the increased iron absorption in spite of iron overload.

Blood transfusions or treatments with iron are ineffective and aggravate iron overload. Nevertheless, administration of apo-transferrin (or apo-transferrin rich plasma) combined with iron chelation therapy can restore hemoglobin and hepcidin levels, reduce NTBI and prolong survival (115, 116). The hypotransferrinemic (hpx) mice provide an animal model of congenital atransferrinemia. They have < 1% of normal circulating transferrin levels due to a point mutation in a splice donor site of the transferrin gene (117). Survival of these animals after weaning depends on administration of apo-transferrin or blood transfusions.

## Atypical inherited disorders of iron overload

*HMOX1* deficiency (OMIM #141250) has been described in a Japanese pediatric patient who presented with systemic iron overload, growth retardation, endothelial cell injury, asplenia, nephritis and inflammation, and died shortly after diagnosis (118, 119). *HMOX1* encodes the heme catabolic enzyme *heme oxygenase 1*, which is highly expressed in tissue macrophages and plays a crucial role in iron recycling from senescent red blood cells. Hmox1^−/−^ mice exhibit partial prenatal lethality (120). Surviving animals have a shortened life span and display pathological features including iron overload, anemia, defective erythrophagocytosis and inflammation (121). Moreover, they are sensitive to infection, endotoxic shock, sepsis and other stresses (122).

Another form of hereditary iron overload (OMIM #615517) has been attributed to a point mutation in the *FTH1* gene (123), which encodes the H-subunit of ferritin, the iron storage protein. The disease was described in a Japanese family as autosomal dominant. The underlying mechanism remains obscure and these findings have not been validated in follow up studies.

More recently, mutations in the *BMP6* gene affecting the processing of the precursor BMP6 pro-peptide to mature BMP6, were linked to an autosomal dominant form of mild hemochromatosis due to hepcidin deficiency (124–126). In light of the previously established iron overload phenotype of Bmp6^−/−^ mice (81, 82) these findings appear plausible. However, causality between the identified BMP6 variants and iron overload is currently controversial (127–129).

## Conclusions

Inherited disorders of iron overload develop as a result of disruption of systemic iron homeostasis. Their genetic and clinical features are summarized in Table 1. The most common is hereditary hemochromatosis, an endocrine disorder of hepcidin deficiency due to various genetic etiologies, leading to diverse clinical phenotypes. The hallmarks of all types of hereditary hemochromatosis are hyperferremia and iron overload in tissue parenchymal cells, with concomitant failure of tissue macrophages and intestinal enterocytes to retain iron. The standard of care for patients involves therapeutic phlebotomy, but hepcidin replacement could provide an etiologic cure.

**Table 1:** Genetic and clinical features of inherited disorders of iron overload.

**Disease name**	**OMIM**	**Gene**	**Locus**	**Transmission**	**Pathology**	**Laboratory features**	**Main clinical expression**
HFE hereditary hemochromatosis	235200	*HFE*	6p21.3	Recessive	Hepatocellular iron loading	↑ serum ferritin and transferrin saturation	Hepatic
HJV juvenile hemochromatosis	602390	*HJV*	1q21	Recessive	Hepatocellular iron loading	↑↑ serum ferritin and transferrin saturation	Cardiac and endocrine
HAMP juvenile hemochromatosis	613313	*HAMP*	19q13	Recessive	Hepatocellular iron loading	↑↑ serum ferritin and transferrin saturation	Cardiac and endocrine
TfR2 hereditary hemochromatosis	604250	*TFR2*	7q22	Recessive	Hepatocellular iron loading	↑ serum ferritin and transferrin saturation	Hepatic
Ferroportin hemochromatosis	606069	*SLC40A1*	2q32	Dominant	Hepatocellular iron loading	↑↑ serum ferritin and transferrin saturation	Hepatic
Ferroportin disease	606069	*SLC40A1*	2q32	Dominant	Mainly Kupffer cell iron loading	↑↑ serum ferritin, normal to low transferrin saturation	Hepatic
Congenital aceruloplasminemia	604290	*CP*	3q23-q24	Recessive	Mainly Kupffer cell iron loading, CNS iron loading	↑ serum ferritin and normal to low transferrin saturation	Neurologic
Congenital atransferrinemia	209300	*TF*	3q22.1	Recessive	Hepatocellular iron loading	↑ serum ferritin and transferrin saturation	Hepatic and hematologic

Ferroportin-associated hemochromatosis is caused by hepcidin resistance and shares phenotypic hallmarks with other forms of hereditary hemochromatosis but is uniquely transmitted in an autosomal dominant manner. Ferroportin disease is a distinct autosomal dominant disorder, often confused with ferroportin-associated hemochromatosis, which is characterized by low transferrin saturation and tissue iron overload predominantly in non-parenchymal cells.

Aceruloplasminemia and atransferrinemia are rare disorders caused by deficiencies in ceruloplasmin or transferrin, respectively. Aceruloplasminemia shares some phenotypic hallmarks of ferroportin disease but is mostly characterized by neurological complications due to brain iron overload, which is unique among all inherited disorders of iron overload. The standard of care for patients involves ceruloplasmin and apo-transferrin replacement therapy, often combined with iron chelation.

## Author contributions

The author confirms being the sole contributor of this work and has approved it for publication.

### Conflict of interest statement

The author declares that the research was conducted in the absence of any commercial or financial relationships that could be construed as a potential conflict of interest.
